# Comparison of Bisulfite Pyrosequencing and Methylation-Specific qPCR for Methylation Assessment

**DOI:** 10.3390/ijms21239242

**Published:** 2020-12-03

**Authors:** Loretta De Chiara, Virginia Leiro-Fernandez, Mar Rodríguez-Girondo, Diana Valverde, María Isabel Botana-Rial, Alberto Fernández-Villar

**Affiliations:** 1 Department of Biochemistry, Genetics and Immunology, University of Vigo, 36310 Vigo, Spain; dianaval@uvigo.es; 2 Centro de Investigaciones Biomédicas (CINBIO), Centro Singular de Investigación de Galicia, Universidad de Vigo, 36310 Vigo, Spain; 3 Pulmonary Department, Hospital Álvaro Cunqueiro, EOXI Vigo, 36213 Vigo, Spain; virginia.leiro.fernandez@sergas.es (V.L.-F.); maria.isabel.botana.rial@sergas.es (M.I.B.-R.); alberto.fernandez.villar@sergas.es (A.F.-V.); 4 PneumoVigo I +i Research Group, Sanitary Research Institute Galicia Sur (IIS Galicia Sur), 36213 Vigo, Spain; 5 Department of Medical Statistics and Bioinformatics, Leiden University Medical Center, 2300RC Leiden, The Netherlands; m.rodriguez_girondo@lumc.nl

**Keywords:** DNA methylation, bisulfite pyrosequencing, methylation-specific qPCR, cut-off, methylation biomarker, non-small cell lung cancer, cytological lymph node samples

## Abstract

Different methodological approaches are available to assess DNA methylation biomarkers. In this study, we evaluated two sodium bisulfite conversion-dependent methods, namely pyrosequencing and methylation-specific qPCR (MS-qPCR), with the aim of measuring the closeness of agreement of methylation values between these two methods and its effect when setting a cut-off. Methylation of tumor suppressor gene *p16/INK4A* was evaluated in 80 lung cancer patients from which cytological lymph node samples were obtained. Cluster analyses were used to establish methylated and unmethylated groups for each method. Agreement and concordance between pyrosequencing and MS-qPCR was evaluated with Pearson’s correlation, Bland–Altman, Cohen’s kappa index and ROC curve analyses. Based on these analyses, cut-offs were derived for MS-qPCR. An acceptable correlation (Pearson’s R2 = 0.738) was found between pyrosequencing (PYRmean) and MS-qPCR (NMP; normalized methylation percentage), providing similar clinical results when categorizing data as binary using cluster analysis. Compared to pyrosequencing, MS-qPCR tended to underestimate methylation for values between 0 and 15%, while for methylation >30% overestimation was observed. The estimated cut-off for MS-qPCR data based on cluster analysis, kappa-index agreement and ROC curve analysis were much lower than that derived from pyrosequencing. In conclusion, our results indicate that independently of the approach used for estimating the cut-off, the methylation percentage obtained through MS-qPCR is lower than that calculated for pyrosequencing. These differences in data and therefore in the cut-off should be examined when using methylation biomarkers in the clinical practice.

## 1. Introduction

Epigenetic mechanisms play an important role in the regulation of gene activity and expression. Aberrant DNA methylation is the most extensively studied epigenetic alteration and is crucial for cancer initiation and development [1]. DNA hypermethylation appears mainly in CpG islands located in the promoter of tumor suppressor genes or DNA repair genes, resulting in their inactivation. In lung cancer, hypermethylation of promoters of well-known cancer-related genes has been reported in many studies [2,3,4]. Aberrant methylation has been detected in liquid biopsy, including blood, sputum, bronchial washings, bronchoalveolar lavage, bronchial aspirates and cytohistological material from endobronchial ultrasound transbronchial needle aspiration (EBUS-TBNA) [4,5,6]. EBUS-TBNA is a minimally invasive method that allows the sampling of mediastinal and hilar lymph nodes for diagnosis and staging of lung cancer [7]. Samples obtained by EBUS-TBNA are typically analyzed using cytological techniques, though molecular markers, including epigenetics, are considered very promising for the detection of occult lymph node metastasis [6,7,8].

The methylation status of the tumor suppressor gene *p16/INK4A* was analyzed in this study, since it has been widely studied in lung cancer (reviewed in [9,10]). *P16/INK4A*, also known as CDKN2A (cyclin-dependent kinase inhibitor 2A), is a negative regulator of the cell cycle progression. Binding of P16 to CDK4 or CDK6 inhibits active cyclin D complex formation and results in G1 phase arrest. Methylation of the *p16/INK4A* promoter results in the loss of control of the restriction point in the G1 phase, favoring cellular transformation [11,12].

Methods for methylation analysis may focus on profiling the whole epigenome, identifying differentially methylated regions or examining specific genes of interest [13,14,15]. PCR-based methods using sodium bisulfite-treated DNA are extensively used for assessing methylation at single loci. Such is the case of pyrosequencing and methylation-specific qPCR (MS-qPCR). Pyrosequencing is an absolute method that provides a quantitative measure of DNA methylation levels at single CpG resolution, determined from the intensity ratio of T and C, the results of which are accurate and reliable for the analysis of short DNA stretches (usually <150 bp) [16]. On the other hand, MS-qPCR is a relative method that measures DNA methylation by comparing samples to a suitable reference, providing information on the methylation status of the region analyzed.

The aim of this study was to measure the closeness of agreement of methylation values between pyrosequencing and nested MS-qPCR, analyzing the methylation status of *p16/INK4A* using cytological samples obtained by EBUS-TBNA in lung cancer patients. Though we found an acceptable correlation between both methods, the estimated cut-off for MS-qPCR was much lower than that derived from pyrosequencing, indicating that these differences should be considered when analyzing methylation in clinical practice.

## 2. Results

### 2.1. p16/INK4A Methylation Analysis Based on Bisulfite Pyrosequencing

**Figure 1:**
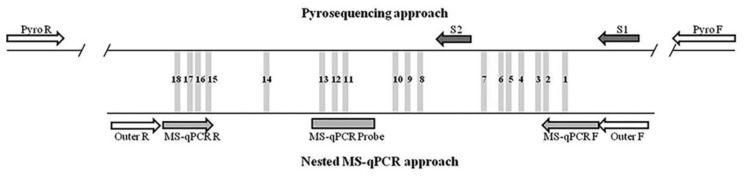
Representation of the CpG island analyzed in *p16/INK4a* promoter. The 18 CpG sites analyzed by pyrosequencing and MS-qPCR are represented as grey bars and are numbered from 1 to 18 in the reverse strand. Above, the design used for the pyrosequencing approach, including PCR primers (Pyro F and Pyro R) and sequencing primers (S1 and S2). Below, the design of the nested-qPCR approach, including PCR outer primers (Outer F and Outer R), qPCR primers (MS-qPCR F and MS-qPCR R) and probe (MS-qPCR Probe).

**Figure 2:**
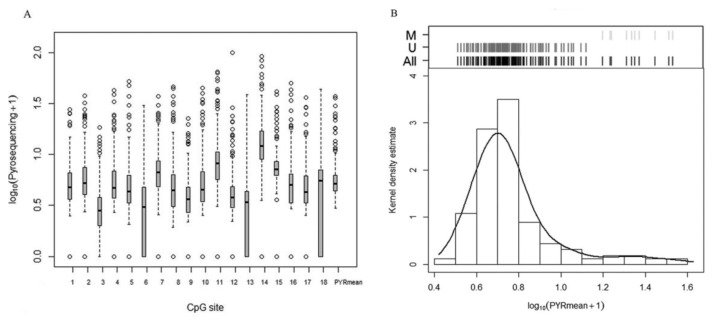
Methylation analysis based on pyrosequencing. (**A**) Boxplots for the distribution of methylation percentage expressed as (log10 (Pyrosequencing + 1)) of each CpG site and the mean pyrosequencing methylation percentage (PYRmean). (**B**) Histogram and kernel density estimation of the distribution of PYRmean expressed as (log10(PYRmean + 1)) used to establish a natural cut-off of methylation percentage. On top of the graph, the corresponding classification graph is shown. Black lines: all samples; dark grey lines: unmethylated (U) group; light grey lines: methylated (M) group.

Eighteen CpG sites from a CpG island in *p16/INK4a* promoter were analyzed using pyrosequencing (Figure 1). Pyrosequencing data were not available for three samples; therefore, these cases were excluded (*n* = 157). Correlation analysis between each CpG site resulted in statistically positive Pearson’s correlation coefficients, ranging from 0.274 to 0.863 (median Pearson’s coefficient 0.620), indicating a considerable level of variability across the CpG sites, as shown in Figure 2A. However, the comparison of each CpG site with the mean pyrosequencing methylation (PYRmean) revealed high linear correlation (median Pearson’s coefficient 0.803, range 0.635–0.917). Therefore, we assumed that PYRmean is a representative measure for pyrosequencing. The boxplot in Figure 2A also includes the PYRmean (median PYRmean 4.21%, range 1.99–36.46%).

The distribution of PYRmean is shown in Figure 2B as histogram and kernel density estimation. According to the cluster analysis, PYRmean data distribution showed the presence of two different groups corresponding to 4.16% and 22.53% medians (*p*-value < 0.001), which suggests the choice of the natural cut-off of 12.54% to define the binary categories of methylated (>12.54%) and unmethylated (≤12.54%). Hence, 10 samples (6.37%) were considered methylated, while the remaining 147 (93.63%) were defined as unmethylated. Table 1 summarizes the distribution of PYRmean as a binary data set according to the presence of metastasis. *p16/INK4A* PYRmean presents 100% specificity (the 10 samples identified as methylated correspond to metastatic samples) and 12.99% sensitivity for detecting metastasis (including both TP and FN samples).

### 2.2. p16/INK4A Methylation Analysis Based on MS-qPCR

**Figure 3:**
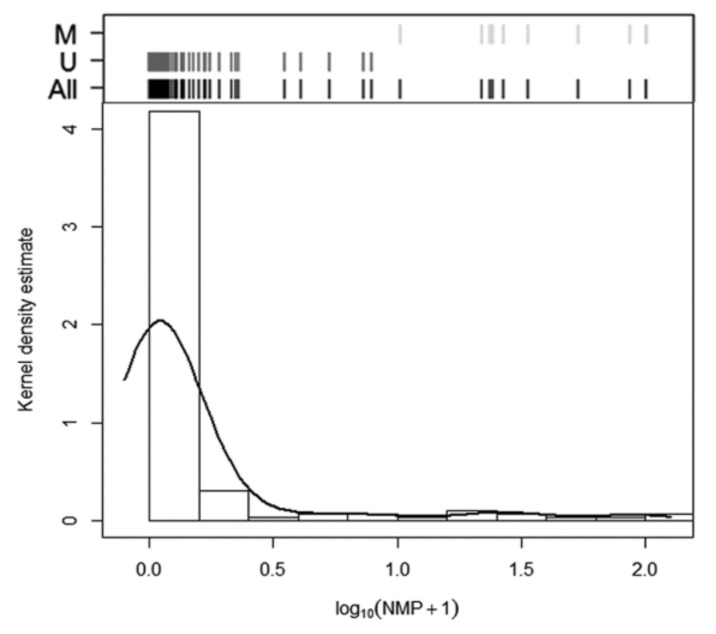
Methylation analysis using MS-qPCR. Histogram and kernel density estimation of the distribution of NMP expressed as (log10(NMP + 1)). The Classification graph based on the natural cut-off derived from cluster analysis is shown above the histogram. Black lines: all samples; dark grey lines: unmethylated (U) group; light grey lines: methylated (M) group.

No amplification was detected in 15 samples; therefore, these samples were excluded (*n* = 145). NMP (normalized methylation percentage) values had a median of 0.089% (range: 9 × 10^−7^ − 100%), showing a skewed distribution towards 0% methylation (Figure 3).

The cluster analysis based on the NMP distribution identified three groups (median NMP 0.08%, 22.95% and 93.04%, respectively), fully distinguished by two cut-off points at 6.86% and 32.56%. Given that the presence of the third group (second cut-off) was due to extremely high methylation percentages detected in only four samples, we merged the second and third groups. Accordingly, a sample was considered methylated when NMP was >6.86% and unmethylated when methylation was ≤6.86%. As in the pyrosequencing analysis, 10 samples (6.90% of the 145 valid samples) were found methylated and 135 (93.10%) unmethylated. This data related to metastatic lymph nodes are shown in Table 1, resulting in 12.86% sensitivity for the detection of micrometastasis, with 98.67% specificity. While pyrosequencing (PYRmean) and MS-qPCR (NMP) provided similar clinical results in terms of sensitivity and specificity when data were categorized as binary using cluster analysis, the samples identified as methylated for each method were not the same (only 5 of 10 samples coincided).

### 2.3. Comparison of the Methylation Approaches for the Study of p16/INK4A

Since methylation analysis of the same region was assessed using two different methods, a direct comparison was performed. This comparison was based on 142 samples for which both methods provided valid results.

**Figure 4:**
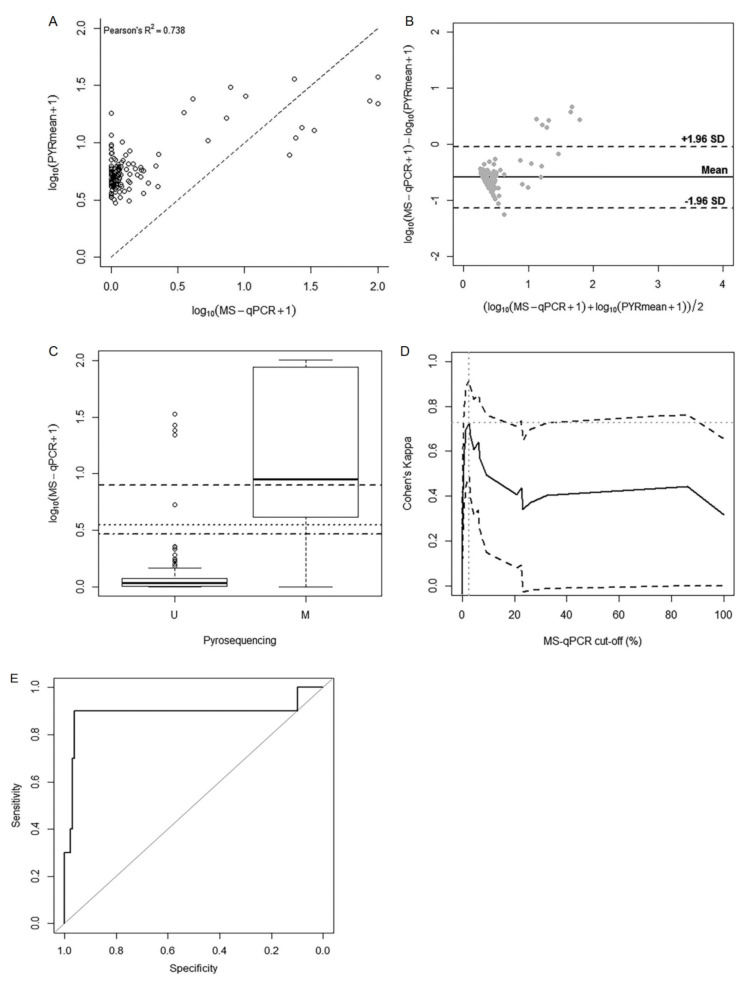
Analysis for the comparison between pyrosequencing and MS-qPCR. (**A**) Scatterplot for the correlation between PYRmean and NMP. (**B**) Bland–Altman data plot used to analyze the agreement between PYRmean and NMP. Black line represents the mean difference between methods, and the discontinue lines represent the 95% limits of agreement. (**C**) Box-plot representing the performance of the NMP methylation percentages with relation to the methylated and unmethylated subgroups derived from the natural cut-off of PYRmean (12.54%). Horizontal lines show the natural cut-off according to the cluster analysis (6.86%; solid line), the cut-off according to the maximization of kappa (2.53%; dashed line) and the cut-off according to the ROC curve analysis (1.90%; dotted line). (**D**) Cohen’s kappa curves for possible MS-qPCR cut-off points to analyze the agreement with pyrosequencing; (**E**) ROC curve analysis of the MS-qPCR (NMP) data to predict the dichotomous classifier based on pyrosequencing (PYRmean).

A good level of correlation (Pearson’s R^2^ = 0.738) was found when PYRmean was compared to NMP. The scatterplot (Figure 4A) representing the association between both methods showed a general tendency of NMP towards more extreme methylation percentages together with more decreased methylation levels than the corresponding PYRmean values. This phenomenon is reinforced by the Bland–Altman plot shown in Figure 4B. Since bisulfite pyrosequencing is considered as a reference, for methylation values below 15%, pyrosequencing showed higher values compared to MS-qPCR, indicating that the latter underestimates methylation. However, for methylation levels above 30%, MS-qPCR showed higher values than pyrosequencing, resulting in overestimation. Moreover, we detected a small group of seven samples falling outside the 95% limits of agreement of the Bland–Altman plot, resulting in considerably higher NMP values compared to PYRmean values, corresponding to metastatic samples in all the cases.

According to the methylation-specific primers and probe binding sites (CpG 1–2, 11–13, 15–18), the correlation analysis revealed similar results (Pearson’s coefficient R^2^ = 0.686). Therefore, the assumption of considering NMP for the 18 CpG sites seems suitable.

When using the proportions of methylated versus unmethylated samples according to the cluster analysis independently conducted for pyrosequencing and MS-qPCR, we obtained a kappa coefficient of 0.492, which provided a barely moderate level of agreement between both binary data based on pyrosequencing of only 50%.

Since pyrosequencing was used as reference, we studied the performance of MS-qPCR using the natural cut-off derived from pyrosequencing (12.54%), considering such dichotomization as optimal. The distribution of NMP in each subgroup was represented in a box-plot (Figure 4C). The difference in the distribution of NMP between subgroups was statistically significant (*p*-value < 0.001). The NMP median value for the unmethylated and methylated subgroup was 0.08% (range 0.00–32.56%) and 8.04% (range 0.00–100%), respectively. However, it is noteworthy that though the pyrosequencing unmethylated group mainly corresponded to low NMP values, Figure 4C shows the existence of a small subset of samples with high methylation levels based on MS-qPCR, consistent with the findings in terms of agreement given by the Bland–Altman plot. On the other hand, the large box in the pyrosequencing methylated group indicated that MS-qPCR data presented high variability in its distribution within this group.

Given the low sensitivity of the original natural cut-off for NMP (6.86%) to detect pyrosequencing methylation, we explored two alternative strategies. Firstly, we derived a new optimal cut-off based on the Cohen’s kappa values. These values corresponded to each of the possible cut-offs based on NMP values to measure the agreement between each one of them and the pyrosequencing measurement. According to this approach, the optimal NMP cut-off for the maximal agreement between methods (maximal kappa index) resulted in 2.53% (Figure 4D). This corresponded to a kappa index of concordance of 0.728, indicating a good level of agreement between both techniques. Moreover, in terms of accuracy to predict methylation according to pyrosequencing, this new cut-off for MS-qPCR entailed better sensitivity than the original natural 6.86% cut-off (80% vs. 50%), maintaining the level of specificity (96%).

Alternatively, we computed the area under the receiver-operating characteristic (AUC) of NMP to predict the dichotomous reference classifier (methylated if PYRmean >12.52% and unmethylated if PYRmean ≤12.52%). Figure 4E shows the resulting ROC curve, with an AUC of 0.876 (95% CI 0.684–1.000). Accordingly, the optimal cut-off that maximizes sensitivity and specificity of the NMP to predict PYRmean was 1.90%, corresponding to a sensitivity of 90% and a specificity of 96%.

These results suggest that for sake of agreement with pyrosequencing in terms of methylation proportions, the cut-off for NMP should be much lower than that derived from pyrosequencing and lower than the cut-off naturally derived from the distribution of NMP.

## 3. Discussion

In this study, we assessed DNA methylation using two sodium bisulfite-based methods. Methylation data for 18 CpG sites in *p16/INK4A* were obtained by both pyrosequencing and MS-qPCR. Data was consistently compared considering pyrosequencing as reference. It is worth mentioning that the techniques compared in our study provide data of a different nature, even though both are continuous scales. Pyrosequencing values come from the mean of 18 CpG sites, resulting in more stable methylation values given its single calculation origin.

In general, our study showed that methylation measurements based on MS-qPCR and pyrosequencing have good correlation (Pearson’s R^2^ = 0.738) when the latter is expressed as PYRmean. The association and agreement between both assays according to Bland–Altman indicated that DNA methylation assessment by MS-qPCR results in an underestimation of methylation below 15%, while methylation greater than 30% appeared overestimated. This overestimation was more evident for seven samples (4.93%), and metastasis was confirmed in all these cases.

The tendency of extreme methylation values has also been described by others. Ogino et al. [17] analyzed MGMT, MLH1 and *p16/INK4A* and observed that the percentage of methylated reference (PMR) for the majority of tumors was low or elevated. The comparison of MS-HRM (methylation-specific high-resolution melting) with pyrosequencing showed a high correlation based on 7 CpG sites in *p16/INK4A* [18]. However, MS-HRM yielded lower methylation values with less methylated samples and higher methylation values with more heavily methylated samples, with 3% of the samples resulting in extremely high methylation values. These differences in methylation may be related to the inclusion of certain CpG sites that favor the binding and amplification of the methylated template over the unmethylated, depending on the methylated/unmethylated template ratio. NGS-based methods like bisulfite amplicon sequencing and enrichment bisulfite sequencing also show extreme values of 0 and 100% more frequently than other assays [19].

Surprisingly, despite the differences observed between both methods, similar clinical results were obtained when methylation data was categorized as binary data sets according to the cluster analysis. Besides cluster analysis, other statistical tools like maximizing the agreement between methods (kappa index) or the ROC curve were used to establish a cut-off for MS-qPCR data. Our results indicate that independently of the method used for estimating the cut-off, the methylation percentage (NMP) cut-off was lower than that calculated for pyrosequencing.

The variability observed in studies examining the efficiency of a biomarker is commonly related to differences in ethnics, age and tumor stage. In addition, differences in sampling, DNA extraction, DNA input and bisulfite modification, primer design, annealing temperature and single-copy genes as reference for normalization in MS-qPCR, together with storage of bisulfite-modified samples may lead to different results [20]. Among the potential sources of variability analyzed, these authors found that the reference gene used for normalization, ALU, COL2A1 or ACTB, caused the largest PMR differences. As described for SFRP2 [21] and GSTP1 [22], different amplicons/primers originate variable results, reflecting the methylation status more or less precisely. According to several considerations concerning potential sources of variations [17,20,23], firstly we prepared a large quantity of fully-methylated control that was use throughout the study. Fully methylated and unmethylated controls were always included in each bisulfite modification. Additionally, dilutions of the fully-methylated control (100%, 10% and 1%) were included in all plates to verify coherent and comparable Cqs between plates. A 1/10 dilution (10%) of the fully-methylated control was included in the pre-amplification step as internal control to verify similarity with the 10% prepared from the amplified fully-methylated control.

It should also be noted that the primers and probe used for MS-qPCR were also used in many other studies evaluating methylation in *p16/INK4A* [24,25,26], increasing the interest in conducting a comparison with a high-resolution, absolute DNA methylation method such as pyrosequencing. This technique has several built-in quality controls and is based on methylation-independent amplification, providing information at single CpG resolution [23]. Another advantage of this technique is the possibility of including a non-methylated control, which allows us to evaluate the performance of the sodium-bisulfite conversion kit used. Pyrosequencing, together with bisulfite amplicon NGS sequencing, were found the best all-round performance methods for methylation marker validation and development according to the study performed by Bock et al. [19] and Šestáková et al. [27] comparing eight and four methylation techniques, respectively. Though absolute methylation assays such as Amplicon BS, enriched BS and pyrosequencing are the methods of choice for biomarker validation, relative methylation assays (MS-HRM or MS-qPCR) with an adequate design are capable of detecting minimal traces of methylated DNA against an excess of unmethylated DNA [19].

Relative quantification is an intrinsic disadvantage of the MS-qPCR method, with the use of probes reducing sensitivity (more restrictive priming) while specificity is increased [17]. However, nested two-stage PCR increases sensitivity, necessary for samples with very low DNA concentration and/or substantially contaminated with DNA from healthy cells. Compared to MethyLight dPCR (digital PCR), Redshaw et al. [14] reported lower variability between replicates for MethyLight qPCR measurements of p14/ARF, with inferior precision to resolve methylation percentages below 50% difference when using the former technique. However, a methylation-independent primer/probe design comparing dPCR and qPCR demonstrated a linear and accurate detection over the complete methylation range, also with low DNA input samples in the case of dPCR [28].

In our study, the differences in methylation between pyrosequencing and MS-qPCR could also be attributed to partial and heterogeneous methylation patterns, which have been described in several tumors [21,29,30]. Quillien et al. [29] suggested that MS-qPCR assays analyzing tumors bearing heterogeneous methylation could result in underestimation because of the restrictive priming increased by the use of a probe.

Though methylation of *p16/INK4A* is of considerable interest in lung cancer, with methylation frequencies ranging from 17 to 80% in tumor tissue and 0 to 80% in plasma, sputum and bronchoalveolar lavage [11], here we found that *p16/INK4A* methylation has no sensitivity for the detection of metastasis in lymph node samples from lung cancer patients, though this was not the aim of the study.

In conclusion, we found that independently of the statistical method used for estimating the cut-off, the methylation percentage obtained through MS-qPCR (NMP) is lower than that calculated for pyrosequencing. The translation of methylation assays of a candidate biomarker to the clinic should take into account the differences and similarities of data obtained using each of the methods, balancing the pros and cons in terms of discriminatory capacity, cost-effectiveness and feasibility of implementation, among others.

## 4. Materials and Methods

### 4.1. Subjects

The study included 80 patients (69 men and 11 women, mean age 62.2) diagnosed of or with high suspicion of NSCLC (non-small cell lung cancer), in which an EBUS-TBNA was performed for diagnostic and/or staging purposes, obtaining a total of 160 lymph node samples. Clinical characteristics of patients and adenopathies are summarized in Table S1. A BF-UC180F-OL8 bronchoscope (Olympus, Japan) and a ProSound alpha 5 ultrasound (Aloka, Japan) were used to examine and puncture adenopathies. An expert pathologist analyzed in situ the cytological lymph node samples recovered. According to cytology and follow-up, samples were classified as 66 (41.25%) true-positives (TP, positive cytology and clinically confirmed metastasis); 82 (51.25%) true-negatives (TN, negative cytology and no evidence of metastasis after surgery or no modification in lymph node size during 1 year surveillance); and 12 (7.50%) false-negatives (FN, negative cytology but metastatic lymph node infiltration evidenced after surgery or significant growth of lymph node detected during follow-up). A representative portion of the sample was resuspended in saline solution and immediately stored at −20 °C.

All patients were monitored at the Pulmonary Department from Hospital Álvaro Cunqueiro (Vigo, Spain). The study followed the clinical-ethical practices of the Spanish Government and the Helsinki Declaration, and it was approved by the Galician Ethical Committee for Clinical Research (CEIC 2009/133). Written informed consent was obtained from each subject and anonymity was warranted.

### 4.2. DNA Extraction and Sodium Bisulfite Modification

DNA was extracted from cytological lymph node samples with QIAamp DNA Blood Mini Kit (Qiagen, Hilden, Germany) and was stored at −20 °C until used. Sodium bisulfite modification was performed using EZ DNA Methylation-Direct kit (Zymo Research, Irvin, CA, USA) according to manufacturer’s instructions. A fully-methylated control was prepared from DNA extracted from peripheral blood from a control individual and treated with CpG methyltransferase (M.SssI; New England Biolabs, Ipswich, MA, USA). An unmethylated control, not treated with M.SssI, was included in each sodium bisulfite treatment.

### 4.3. Bisulfite Pyrosequencing

Methylation analysis at single CpG sites was performed targeting a CpG island located in the promoter of *p16/INK4A* (Figure 1). Each amplification/sequencing set included a fully-methylated control, an unmethylated control and a no template control (NTC). The PCR and sequencing primers (Table S2) were designed with the Pyromark Assay Design software v2.0 (Qiagen). PCR was performed in 25 µL containing 3 µL of bisulfite-treated DNA, 0.72 µM forward and reverse primers, 75 µM dNTPs, 1X Ex Taq Buffer and 1 unit Takara Ex Taq HotStart, with the following cycling conditions: 95 °C for 5 min, 38 cycles of 95 °C for 30 s, 62 °C for 30 s and 72 °C for 30 s and, finally, 72 °C for 7 min. A 391 bp region was amplified (Chromosome 9, genomic coordinate 21,974,599–21,974,990). To verify amplification, 5 µL of PCR product were run on a 3% agarose gel. Single-stranded biotinylated PCR products were prepared for sequencing with the PyroMark TM Vacuum Prep Workstation (Qiagen, Hilden, Germany).

Two sequencing reactions were carried out to interrogate the 18 CpG sites (Figure 1; first reaction CpG 1–7, second reaction CpG 8–18). A PyroMark MD instrument and the Pyro Q-CpG software (Qiagen, Hilden, Germany) were used. The mean methylation percentage (PYRmean) was calculated by averaging across all CpG sites from both sequencing reactions.

### 4.4. Methylation-Specific qPCR (MS-qPCR)

Methylation of the same CpG island in *p16/INK4A* was analyzed using a nested methylation-specific qPCR (Figure 1). In the first PCR (pre-amplification step), a 193 pb methylation-independent product was amplified (Chromosome 9, genomic coordinate 21,974,736–21,974,929) using outer primers [24] (Table S1). PCR was performed in 25 µL containing 3 µL of bisulfite-treated DNA and the previously described reaction mix, with the following cycling conditions: 95 °C for 5 min, 32 cycles of 95 °C for 30 s, 64 °C for 30 s and 72 °C for 30 s and, finally, 72 °C for 7 min. A fully-methylated control, an unmethylated control and an NTC were always included in each PCR. In addition, a 1/10 and 1/100 dilution of the methylated control were also included (referred to PCR tube A and PCR tube B, respectively).

In the second step, a MS-qPCR was performed using a 1/500 dilution of the PCR product obtained in the pre-amplification step. Real-time PCR was carried out in triplicate in 20 µL containing 2 µL of diluted PCR product, 600 nM each primer [25], 200 nM probe [26] and 1X TaqMan Universal PCR Master Mix No AmpErase UNG (Applied Biosystems, Waltham, MA, USA), with an annealing temperature of 60 °C, amplifying a 150 pb product (Chromosome 9, genomic coordinate 21,974,757–21,974,907). Amplifications were run on a StepOne instrument (Applied Biosystems, Waltham, MA, USA). In each plate, dilutions of the pre-amplified fully-methylated control, unmethylated control, samples and NTC were included, besides MS-qPCR NTC.

Additionally, to verify that the nested approach did not introduced changes in methylation levels, in each qPCR plate we included the following: a 1/500 dilution of PCR tube A (10% methylation) and B (1% methylation), besides a 1/500 dilution of a 10% methylation and 1% methylation prepared using the pre-amplified fully-methylated control. In all the runs, the Cq (quantification cycle) values of the same methylation percentage were equivalent, indicating that methylation levels were not affected by the two-step approach.

A region of the MYOD1 gene that lacks any CpG site was used to normalize for DNA input. This assay reflects the amount and integrity of the input genomic DNA [31]. The same two-step nested PCR approach was used for MYOD1, using the primers described in Table S1. The annealing temperature was 60 °C and the product corresponded to 162 pb (Chromosome 11, genomic coordinate 17,714,203–17,714,365).

### 4.5. Analysis of the MS-qPCR Data

MS-qPCR *p16/INK4A* methylation data was derived from 5 independent assays (standard curves) consisting of dilutions of the pre-amplified fully-methylated control (100, 75, 50, 25, 10, 5, 1, 0.5, 0.1% methylation). The non-normalized methylation percentage (NNMP) of each sample was estimated from a linear fit of the mean Cq as a function of the log10 methylation percentage (amplification efficiency: 92.02%; slope: −3.5295; R^2^ = 0.9993). DNA quantity (DNAQ) using MYOD1 was obtained for each sample for normalization (standard curve 91.06% efficiency; slope: −3.5566, R^2^ = 0.9985). Finally, the normalized methylation percentage (*NMP*) was calculated as follows [6]:

$$NMPsample=\frac{NNMPsample\;\left(p16/INK4A\right)}{DNAQsample\;\left(MYOD1\right)}\;\times 100$$

### 4.6. Statistical Analysis

MS-qPCR (NMP) and pyrosequencing (PYRmean) values were log10 + 1 transformed for statistical analysis. Clustering algorithms based on non-parametric density estimation [32] were used to establish two subgroups (methylated and unmethylated) according to each method. Pearson’s correlation was used to measure the association between the two methods. Bland–Altman analysis was conducted to assess agreement, showing the differences in measurements among the methods and any relationship between the differences and the true values. Concordance between methylation subgroups was explored using Cohen’s kappa index, estimating the maximal kappa index which corresponds to maximal agreement between methods. A Mann–Whitney test was used to compare the levels of PYRmean and NMP between the methylated and unmethylated subgroups. We explored two strategies for deriving MS-qPCR subgroups, both relying on the pyrosequencing subgroups as reference. We firstly proposed to maximize the Cohen’s kappa index and secondly, the simultaneous optimization of both sensitivity and specificity across the NMP values. *p*-values < 0.05 were considered as statistically significant. All statistical analyses were carried out with the statistical software R.

## Figures and Tables

**Table 1:** *p16/INK4a* methylation according to pyrosequencing and MS-qPCR based on different statistical analyses.

	METHYLATED *n* (Number of Samples) (Median and Range of Methylation Percentage)				UNMETHYLATED *n* (Number of Samples) (Median and Range of Methylation Percentage)			
	PYRmean *	NMP ^†^	NMP ^‡^	NMP ^§^	PYRmean *	NMP ^†^	NMP ^‡^	NMP ^§^
**All samples**	*n* = 10 22.53 (15.33–36.46)	*n* = 10 29.27 (9.22–100)	*n* = 14 22.95 (3.10–100)	*n* = 15 22.63 (2.52–100)	*n* = 147 4.16 (1.99–12.54)	*n* = 135 0.08 (0.00–6.85)	*n* = 131 0.07 (0.00–2.52)	*n* = 130 0.07 (0.00–1.28)
**Metastatic** **lymph nodes**	*n* = 10 22.53 (15.33–36.46)	*n* = 9 25.97 (9.22–100)	*n* = 12 22.95 (3.10–100)	*n* = 13 22.63 (2.52–100)	*n* = 67 4.17 (2.21–12.54)	*n* = 61 0.09 (0.00–6.85)	*n* = 58 0.09 (0.00–2.52)	*n* = 57 0.08 (0.00–1.28)
**Non-metastatic lymph nodes**	*n* = 0 - -	*n* = 1 52.83 -	*n* = 2 28.58 (4.33–52.83)	*n* = 2 28.58 (4.33–52.83)	*n* = 80 4.10 (1.99–10.35)	*n* = 74 0.06 (0.00–4.33)	*n* = 73 0.06 (0.00–1.24)	*n* = 73 0.06 (0.00–1.24)

* For pyrosequencing (PYRmean), a cut-off >12.54% based on the cluster analysis was used. ^†^ For MS-qPCR (NMP; normalized methylation percentage), a cut-off >6.86% based on the cluster analysis was used. ^‡^ For MS-qPCR (NMP), a cut-off >2.53% was used based on the maximum agreement using kappa index. ^§^ For MS-qPCR (NMP), a cut-off >1.90% was used based on the ROC curve analysis.

## Supplementary Materials

The following are available online at https://www.mdpi.com/1422-0067/21/23/9242/s1.

## Abbreviations

EBUS-TBNA	Endobronchial ultrasound transbronchial needle aspiration
MS-qPCR	Methylation-specific qPCR
NMP	Normalized methylation percentage
NSCLC	Non-small cell lung cancer
PYRmean	Mean methylation percentage
