# Supplementary material for: Comparison of Bisulfite Pyrosequencing and Methylation-Specific qPCR for Methylation Assessment
Source: Int J Mol Sci. 2020 Dec 3;21(23):9242. doi: 10.3390/ijms21239242 (PMC7730915; doi:10.3390/ijms21239242)
Supplement: Supplementary file 1 [file ijms-21-09242-s001.pdf]

Table 1. Clinical characteristics of patients and adenopathies.

**Variable**	**n (%)**
Sex (male)	69 (86.3%)
Age (mean±SD)	62.2±9.73
Tumor stage	
IA	6 (7.5%)
IB	10 (12.5%)
IIA	5 (6.3%)
IIB	15 (18.8%)
IIIA	17 (21.3%)
IIIB	10 (12.5%)
IV	17 (21.3%)
Tumor histology	
adenocarcinoma	52 (65.0%)
squamous cell	25 (31.3%)
large cell	3 (3.8%)
Cytological result	
TP	66 (41.2%)
TN	82 (51.3%)
FN	12 (7.5%)
Lymph nodes per patient	
1	32 (40.0%)
2	27 (33.8%)
3	13 (16.3%)
4	5 (6.3%)
5	3 (3.8%)

Table S2. Primer and probe sequences used for the study of *p16/INK4A* by bisulfite pyrosequencing and methylation-specific qPCR.

Type of Analysis<br>Name	Sequence<br>(5'-3')
Pyrosequencing p16/INK4a	
Pyro F	GGTTTTTTTAGAGGATTTGAGGGATAG
Pyro R	Biotin-TAATTCCAATTCCCCTACAAACTT
S1	GTTGGTTATTAGAGGGTG
S2	AGAGGGGGAGAGTAG
MS-qPCR p16/INK4a	
Outer F [24]	AGAAAGAGGAGGGGTTGGTTGG
Outer R [24]	ACRCCCRCACCTCCTCTACC
MS-qPCR F [25]	TTATTAGAGGGTGGGGCGGATCGC
MS-qPCR R [25]	GACCCCGAACCGCGACCGTAA
MS-qPCR Probe [26]	6FAM-AGTAGTATGGAGTCGGCGGCGGG-MGB
qPCR MYOD1	
Outer F/qPCR F	CCAACTCCAAATCCCCTCTCTAT
Outer R/qPCR R	TGATTAATTTAGATTGGGTTTAGAGAAGGA
qPCR Probe	6FAM-<br>TCCCTTCCTATTCCTAAATCCAACCTAAATACCTCC
